# Age-specific findings on lifestyle and trajectories of cognitive function from the Korean Longitudinal Study of Aging

**DOI:** 10.4178/epih.e2023098

**Published:** 2023-11-02

**Authors:** Seungju Lim, Eunyoung Yoo, Ickpyo Hong, Ji-Hyuk Park

**Affiliations:** 1 Department of Occupational Therapy, Graduate School, Yonsei University, Wonju, Korea; 2 Department of Occupational Therapy, College of Software and Digital Healthcare Convergence, Yonsei University, Wonju, Korea

**Keywords:** Life style, Cognition, Longitudinal studies, Aging, Republic of Korea

## Abstract

**OBJECTIVES:**

Few longitudinal studies have explored age-related differences in the relationship between lifestyle factors and cognitive decline. This study investigated lifestyle factors at baseline that slow the longitudinal rate of cognitive decline in young-old (55–64 years), middle-old (65–74 years), and old-old (75+ years) individuals.

**METHODS:**

We conducted an 11-year follow-up that included 6,189 older adults from the Korean Longitudinal Study of Aging, which is a cohort study of community-dwelling older Koreans. Lifestyle factors, including physical activity, social activity (SA), smoking, and alcohol consumption were assessed at baseline. Cognitive function was measured at 2-year intervals over 11 years. Latent growth modeling and multi-group analysis were performed.

**RESULTS:**

The influence of lifestyle factors on the rate of cognitive decline differed by age. Smoking at baseline (−0.05; 95% confidence interval [CI], −0.11 to −0.00, per study wave) accelerated cognitive decline in young-old individuals, whereas frequent participation in SA at baseline (0.02; 95% CI, 0.01 to 0.03, per study wave) decelerated cognitive decline in middle-old individuals. None of the lifestyle factors in this study decelerated cognitive decline in old-old individuals.

**CONCLUSIONS:**

Cognitive strategies based on modifiable lifestyle factors such as smoking cessation in young-old individuals and frequent SA participation in middle-old age individuals may have great potential for preventing cognitive decline. Because the influence of lifestyle factors varied by age group, age-specific approaches are recommended to promote cognitive health.

## GRAPHICAL ABSTRACT

[Fig f1-epih-45-e2023098]

## INTRODUCTION

Declining cognitive function is a major issue associated with aging [1]. Studies have focused on identifying modifiable lifestyle factors that can preserve cognitive function and prevent cognitive decline in the older population [2]. Lifestyle refers to an individual’s multifaceted health habits [4], including physical activity (PA) [3], social activity (SA) [4], smoking [5], and alcohol consumption habits [6]. Older individuals can maintain their cognitive function during the aging process by adopting healthy lifestyles. According to the “use it or lose it” hypothesis, unhealthy lifestyle behaviors, including sedentary habits, social disengagement, smoking, and alcohol consumption, are related to declining cognitive function compared with healthy lifestyle habits such as exercising, social engagement, non-smoking, and non-drinking [5,6]. However, evidence derived from cross-sectional studies may have a limited ability to support conclusions regarding more complex associations of change [2]. Therefore, longitudinal studies are required to determine the relationship between lifestyle factors and age-related cognitive decline.

Recent longitudinal studies have reported the impact of lifestyle on cognition. PA at baseline has been associated with the rate of change in cognitive function, and physically inactive older adults reportedly experience a greater decline in cognitive function than the reference group [7,8]. A meta-analysis of longitudinal studies demonstrated a favorable relationship between PA and cognitive trajectories during the follow-up period [9,10]. However, the impact of lifestyle factors such as social engagement, smoking, and alcohol consumption on cognitive decline has not been consistently reported. Some studies have reported that SA at baseline may help delay cognitive decline [11,12], whereas others have failed to demonstrate this [13,14]. Similarly, a reciprocal relationship study [15] reported that cognitive function could modify subsequent changes in confiding and practical social support, but not vice versa. Other lifestyle studies have demonstrated that smoking [16] and alcohol consumption [16] have a significant effect on accelerating the rate of decline in cognitive function, whereas another study showed that global cognition was not associated with alcohol consumption at any point over long periods [17]. Some well-designed randomized controlled trials have also failed to discover any significant effects of PA on cognitive function [18–20]. The varied findings of longitudinal studies may be attributed to the heterogeneity of the elderly population.

The older population is not a biologically or socially homogeneous group [21]. The rate of cognitive decline accelerates with age, indicating that the slope of decline is steeper in the oldest-old group than in the young-old group [22]. The young-old group reportedly engages more in SA than the oldest-old group because they usually work and have not yet retired [23]. This multifaceted heterogeneity may influence the effects of lifestyle factors on cognitive decline. Target lifestyle factors for preventing dementia have been reported to change according to the lifespan. Education is a modifiable risk factor for dementia in early life, whereas smoking and social isolation are modifiable risk factors in late life [24]. The risk factors for memory decline differ between the young-old and old-old groups [24]. Fewer self-maintenance activities and lower SA are lifestyle risk factors in the young-old and old-old age groups. These findings suggest that lifestyle factors that prevent cognitive decline are diverse and depend on aging [24]. Although evidence regarding the association between lifestyle factors and cognitive function has been reported, it is insufficient to determine which lifestyle factors decelerate cognitive decline in different stages of aging, such as young-old, middle-old, and old-old age groups. Identifying the key lifestyle factors affecting cognitive decline at different stages of aging may be challenging. Latent growth modeling (LGM) is a powerful tool for analyzing the relationship between lifestyle factors and changes in cognitive decline. Therefore, this study investigated lifestyle factors that slow the rate of cognitive decline in different age groups.

## MATERIALS AND METHODS

### Study sample and procedures

Data were obtained from the second (2008) to seventh waves (2018) of the Korean Longitudinal Study of Aging (KLoSA), a longitudinal study of community-dwelling older adults at the national level. Since 2006, the Korea Labor Institute has collected data on 10,254 community-dwelling older adults aged ≥45 years from all regions of Korea, except Jeju Island, every 2 years, with an average sample retention rate of 77.6% until 2018 [25]. The KLoSA collects information on health status, PA, SA, and health behaviors. The present study extracted data for approximately 11 years from 2008 to 2018 from the KLoSA database. The exclusion criteria were age <55 years at baseline, missing values for cognitive function at any time point, and missing values for lifestyle factors at baseline. The analysis included 6,189 participants in 2008, and the retention rate of these participants was 85.94% in 2010, 93.38% in 2012, 92.45% in 2014, 93.03% in 2016, and 89.47% in 2018. The study participants were classified into 3 groups according to their baseline age: young-old (55–64 years, n=2,346); middle-old (65–74 years, n=2,407); and old-old (75 years or more, n=1,436).

### Cognitive function

The cognitive function of study participants over 11 years (2008–2018) was measured using the Korean version of the Mini-Mental State Examination (K-MMSE) [26]. The original version of the K-MMSE is a validated measure of cognitive function in the Korean population [27]. The K-MMSE measures time/spatial orientation, memory registration, attention and calculation, memory recall, language, and visual construction. The questionnaire includes 19 questions with a maximum score of 30 points. Higher scores indicate better cognitive function. In this study, the estimated Cronbach’s alpha was approximately 0.799.

### Lifestyle factors

The lifestyle factors assessed in this study included PA, SA, smoking status, and alcohol consumption. All lifestyle variables used in this study were measured at baseline (2008). PA at baseline was measured by asking respondents about their regular exercise status and duration. Regular exercise status was assessed by asking participants whether they exercised regularly at least once a week. The responses were dichotomized as 1 (regular exercise) or 0 (no exercise) [2]. The regular exercise duration was assessed by asking respondents how long they had been exercising. The responses were categorized as 0 (none), 1 (<1 year), 2 (1–2 years), 3 (3–4 years), 4 (5–6 years), and 5 (≥7 years) [28]. SA at baseline was measured by inquiring about respondents’ diversity and frequency of SA participation. The diversity of SA participation was assessed by asking if they participated in the following 6 types of SA: (1) religious activities; (2) friendship activities; (3) leisure, culture, or sports clubs; (4) family or school reunions; (5) volunteer work; and (6) political activities. The responses were summed and categorized into 0 (no participation), 1 (one activity), and 2 (≥two activities) [29]. The frequency of SA participation was assessed by asking participants how often they took part in the above activities. Higher scores indicated a higher frequency of SA participation. Alcohol consumption responses were dichotomized as 1 (current drinker) or 0 (never-drinker) [2]. Smoking status responses were also dichotomized as 1 (current smoker) or 0 (never-smoker) [2]. Responses such as “not returned,” “no answer,” “N/A,” and “don’t know” for any of the 6 variables that were being assessed were considered to be missing values and removed.

### Covariates

All covariates used in this study were measured at baseline (2008). Age, sex, educational level, marital status, residence, log-transformed household income, and employment status were measured. The number of chronic diseases was determined by asking participants if they had been diagnosed with specific conditions: hypertension, diabetes, cancer, pulmonary diseases, hepatic diseases, cardiovascular disease, cerebrovascular diseases, psychiatric diseases, and arthritis. The responses were categorized into 3 categories: 0 (none), 1 (1 disease), and 2 (2 diseases or more) [30]. Self-rated health was measured using a 5-point Likert scale with scores ranging from 1 (very good) to 5 (very bad). Higher scores were reverse-coded to indicate higher self-rated health [2]. Depressive symptoms were measured using an adapted version of the Center for Epidemiologic Studies Depression Scale. The scale consisted of 10 items rated on a 4-point Likert scale from 0 (rarely or none of the time) to 3 (most of the time) [31,32]. Each response was then converted into a binary form, with 0 indicating rarely or none of the time, and 1 indicating sometimes, often, or most of the time [31,32]. Higher summed scores indicated more severe depression.

### Statistical analysis

Descriptive statistics were used to examine the demographic characteristics of the study participants. LGM and multi-group analyses (MGA) were performed. LGM consists of unconditional and conditional models. First, an unconditional model was used to examine the initial level (i.e., intercept) and rate of change (slope) of cognitive function. To determine the most accurate model for estimating the cognitive trajectory, we conducted the chi-square difference test between the no-growth and linear slope models. Second, MGA was performed among the young-old, middle-old, and old-old groups to examine age-related differences in cognitive trajectories. Third, after verifying the significance of the variance in the intercept and slope of the cognitive trajectories, we applied a conditional model to examine the association between the initial level and rate of change in cognitive function and lifestyle factors, along with the effect of lifestyle factors on the intercept and slope of cognitive function. Lastly, MGA was performed to examine the impact of age differences on lifestyle factors in the cognitive trajectories. The goodness-of-fit of the model was determined using the comparative fit index (CFI; ≥0.90), Tucker-Lewis index (TLI; ≥0.90), root mean square error of approximation (RMSEA; ≥0.06), and standardized root mean square residual (SRMR; ≤0.08) [33]. The chi-square statistic (χ^2^) was reported in this study but was not used to determine model fit because of its high sensitivity to a large sample size [33]. Missing data were handled using full information maximum likelihood (FIML). Statistical analyses were performed using SPSS version 25.0 (IBM Corp., Armonk, NY, USA), and Mplus 8.8.

### Ethics statement

This study involved human participants and was conducted in accordance with the principles of the Declaration of Helsinki. The KLoSA was approved by the Korea Labor Institute (approval No. 33602). All participants provided informed consent before the study commencement. The study protocol for secondary data analysis was approved by the Yonsei University Institutional Review Board (approval No. 1041849-202005-SB-067-01).

## RESULTS

### Descriptive results

The sample comprised 2,346, 2,407, and 1,436 participants in the young-old, middle-old, and old-old groups, respectively. Analysis of variance revealed significant differences in age among the groups, even after post-hoc analysis. Participants in the young-old age group were highly educated, married, wealthier, self-rated as healthier, and had a lower likelihood of being diagnosed with chronic diseases and experiencing depression (Table 1). In addition, participants in the young-old age group were more likely to exercise regularly, participate in SA, smoke, and consume alcohol (Table 2).

### Estimation of cognitive trajectories

The chi-square difference test was performed between the linear slope and no-growth models to determine the most accurate trajectory shape. Significant differences were observed between the linear slope (χ^2^ [degree of freedom, df]=274.43 [16], p<0.001; CFI=0.99; TLI=0.99; SRMR=0.02; RMSEA=0.05) and no growth (χ^2^ [df]=2,077.72 [19], p<0.001; CFI=0.89; TLI=0.89; SRMR=0.19; RMSEA=0.13) models, suggesting that the linear slope models adequately represented the cognitive trajectories.

An unconditional linear slope model of the LGM was used to identify participants’ cognitive trajectories. Their cognitive trajectories began with a K-MMSE score of 24.32, with a rate of change of −0.20 for a 2-year interval (χ^2^ [df]=274.43 [16], p<0.001; CFI=0.99; TLI=0.99; SRMR=0.02; RMSEA=0.05). The unconditional LGM with MGA differed significantly according to age group (Δχ^2^ [df]=356.10 [48], p<0.001; CFI=0.98; TLI=0.98; SRMR=0.04; RMSEA=0.06), with the sharpest declining pattern in time evident in the old-old group. The significance of the covariance of the intercept and slope was observed in the young-old and middle-old groups (Table 3).

### Effects of lifestyle factors on cognitive trajectories

Conditional LGM was performed to identify the effects of lifestyle factors on the intercepts and slopes of participants’ cognitive function. After adjusting for age group, sex, education, marital status, residence, log transformed household income, employment status, chronic diseases, self-rated health, and depressive symptoms (χ^2^ [df]=482.61 [76], p<0.001; CFI=0.98; TLI=0.97; SRMR=0.01; RMSEA=0.03), frequency of SA participation at baseline decreased the longitudinal cognitive decline by 0.01. However, smoking at baseline accelerated cognitive decline by −0.05 (Table 4). When considering covariates, older age group had a negative effect (−0.14), while high income and high depressive symptoms had positive effects (0.02 and 0.01, respectively) on the rate of cognitive decline.

The conditional LGM with MGA showed significant differences according to age group after adjusting for covariates (Δχ^2^ [df]= 683.52 [240], p<0.001; CFI=0.97; TLI=0.96; SRMR=0.02; RMSEA=0.03). In the young-old group, smoking was associated with accelerated cognitive decline by −0.05 (Table 4). In the middle-old group, frequent participation in SA was the only factor that reduced cognitive decline by 0.02 (Table 4). In the old-old group, none of the lifestyle factors significantly affected cognitive decline (Table 4). When considering covariates, increasing age had a negative effect on cognitive decline in all age groups (young-old: −0.01; middle-old: −0.02; old-old: −0.03). However, being male (0.07), living in a city (0.04), having higher income (0.02), and experiencing high depressive symptoms (0.01) showed a positive effect on the rate of cognitive decline in young-old individuals (Table 4).

## DISCUSSION

This study was the first to analysis the age-specific effects of lifestyle factors on the cognitive trajectory of the Korean older adults during an 11-year period, using LGM and MGA. This study examined lifestyle factors at baseline that slowed the rate of cognitive decline in different age groups. The results revealed differences in the influence of lifestyle factors on the cognitive decline rate with age. Smoking at baseline accelerated cognitive decline in the young-old group, whereas frequent SA participation at baseline delayed cognitive decline in the middle-old group. None of the lifestyle factors assessed in this study decelerated the cognitive decline in the old-old group.

In the present study, different lifestyle factors affected the rate of cognitive decline in the young-old and middle-old groups. The results revealed that smoking at baseline was associated with accelerated cognitive decline in the young-old group, whereas frequent participation in SA at baseline was associated with slower cognitive decline in the middle-old group. This finding is consistent with that of a previous study reporting a faster decline in global cognition among individuals in the young-old group who smoked [34]. In the middle-old group, only the frequency of SA participation, not its diversity, slowed cognitive decline. A previous longitudinal systematic review and meta-analysis [35] confirmed that frequent social interactions are important for preventing cognitive decline in middle-old individuals. Although the effects of the diversity and frequency of SA participation on cognitive decline have been widely discussed [36], recent studies have reported that frequency is more sensitive than diversity in predicting cognitive abilities [37]. In particular, individuals in the middle-old group have more time to engage in SA than those in the young-old group because they have retired [23]. The social and biological heterogeneity of the age groups may have resulted in the different effects of lifestyle factors on cognitive decline. In addition, the findings in the old-old group differed from those in the young and middle-old groups.

We did not identify a significant association between lifestyle factors and cognitive decline in the old-old group, contrary to the findings of previous studies. Some modifiable lifestyle factors (including PA, SA, and health behaviors) have been reported to be predictors of cognitive trajectories associated with slower cognitive decline [38], even in individuals aged >75 years. However, the race and ethnicity of individuals comprising the populations in previous studies included in the systematic review differed from those in Korean population-based studies. Racial and ethnic differences in the study population may explain why lifestyle factors were not associated with cognitive decline in the old-old group. In addition, the KLoSA data used in this study are considered to be representative in reflecting the cultural and environmental characteristics of Korean individuals belonging to the old-old group through systematic sampling with a probability proportional to size [25].

Contrary to previous studies, we found that the level of PA was not associated with a decreased rate of cognitive decline throughout the aging stages. A meta-analysis of prospective cohort studies [10,39] reported that PA protects against a higher rate of cognitive decline. However, the limitations of the study, including the mix of PA components (i.e., housework, gardening, cleaning the car), the limited sample populations of both sexes (male/female), and age (aged 65 years), should also be acknowledged. Few studies have focused on regular exercise, a representative measurement variable of PA, rather than on overall PA. Because the present study focused only on exercise and not on PA in a more general sense, the results regarding the influence of PA on cognitive decline might differ from those of previous studies [40].

This study has a few limitations. First, the variable “regular exercise duration” measured the period of regular exercise until the baseline (2008). Therefore, exercise periods that ended before the survey or those that occurred after the survey were not considered in this study. Second, this variable was highly skewed toward the value of 0, which was found in 58.82% of participants in the young-old group, 65.02% in the middle-old group, and 76.95% in the old-old group, compared to the values from 1 to 5. This imbalance in the data may result in sample shortages and biased distributions. Therefore, interpreting the results in a statistically significant and generalizable manner should be done with caution. In addition, we must consider the limitations of the measurement accuracy of overall PA using a questionnaire. Further longitudinal studies including both sexes beginning in the young-old or earlier age groups can help resolve the unanswered hypothesis regarding the relationship of PA with longitudinal cognitive decline. Third, this study included time-invariant lifestyle factors measured at baseline. Future studies should consider time-varying lifestyle factors to identify dynamic longitudinal associations between changes in lifestyle factors and cognitive decline. Further studies are also required to determine the dynamic associations between specific cognitive domains and lifestyle factors in different age groups.

Despite these limitations, this study holds significant value as it is the first to investigate the age-specific effects of lifestyle on longitudinal cognitive decline among young-old, middle-old, and old-old groups in a population-based Korean sample. Throughout the 11-year follow-up period, this study revealed distinct lifestyle factors that contributed to delaying the rate of cognitive decline in each age group. Frequent participation in SA was found to delay cognitive decline among the middle-old, while smoking was associated with the acceleration of cognitive decline in the young-old. For instance, smoking cessation among the young-old and frequent participation in SA among the middle-old were found to have potential in preventing cognitive decline. These findings highlight the importance of modifiable lifestyle factors in promoting cognitive health, and the study sheds light on the significance of age-specific approaches in cognitive health interventions.

In conclusion, this study has provided valuable insights into the relationship between lifestyle factors and cognitive decline among different age groups. By exploring the effects of smoking and frequent SA participation on cognitive changes over an 11-year period in the young-old, middle-old, and old-old groups, this research reexamined and definitively confirmed the impact of these factors on cognitive health. The identification of age-specific influences emphasizes the necessity for tailored strategies to prevent cognitive decline in various stages of aging. Overall, this study contributes to the growing body of knowledge on cognitive health and highlights the potential benefits of targeted interventions to promote cognitive well-being across different age groups.

## Figures and Tables

**Figure f1-epih-45-e2023098:**
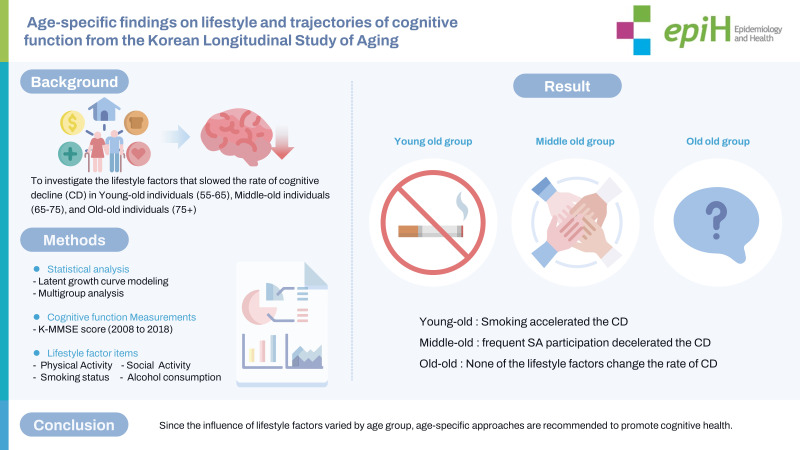


**Table 1.** Demographic characteristics of the participants according to age group

Characteristics		Young-old	Middle-old	Old-old	p-value^1^
Age		59.43±2.82	69.33±2.81	80.29±4.50	<0.001
Sex					<0.001
	Male	1,089 (46.4)	1,063 (44.2)	540 (37.6)	
	Female	1,257 (53.6)	1,344 (55.8)	896 (62.4)	
Level of education					<0.001
	Below elementary	906 (38.6)	1,572 (65.3)	1,171 (81.5)	
	Middle school	537 (22.9)	319 (13.2)	97 (6.7)	
	High school	678 (28.9)	360 (16.0)	112 (7.8)	
	Above college	224 (9.5)	155 (6.4)	56 (3.9)	
Marital status					<0.001
	Married	2,041 (87.0)	1,788 (74.3)	700 (48.7)	
	Other	305 (13.0)	619 (25.7)	736 (51.3)	
Area of residence					<0.001
	Urban	1,811 (77.2)	1,680 (69.8)	974 (67.8)	
	Rural	535 (22.8)	727 (30.2)	462 (32.2)	
Log-transformed household income		2,757.83±3,344.73	1,596.06±2,986.93	1,647.48±1,836.78	<0.001
Employment status					<0.001
	Currently working	1,188 (50.6)	684 (28.4)	125 (8.7)	
	Other	1,158 (49.4)	1,723 (71.6)	1,311 (91.3)	
Chronic diseases					<0.001
	None	1,098 (46.8)	732 (30.4)	371 (25.8)	
	1 disease	758 (32.3)	811 (33.7)	500 (34.8)	
	≥2 diseases	490 (20.9)	864 (35.9)	565 (39.3)	
Self-rated health		3.18±0.87	2.78±0.88	2.51±0.86	<0.001
Depressive symptoms		3.28±2.83	4.22±2.98	5.15±3.98	<0.001

Values are presented as number (%) or mean±standard deviation.

1 Using the chi-square test and analysis of variance for categorical and continuous covariates, respectively.

**Table 2.** Lifestyle characteristics of the participants according to age group

Characteristics		Young-old	Middle-old	Old-old	p-value^1^
Regular exercise status					<0.001
	Yes	966 (41.2)	842 (35.0)	331 (23.0)	
	No	1,380 (58.8)	1,565 (65.0)	1,105 (76.9)	
Regular exercise duration (yr)					<0.001
	None	1,380 (58.8)	1,565 (65.0)	1,105 (76.9)	
	< 1	115 (4.9)	3.49 (3.5)	33 (2.3)	
	1-2	226 (9.6)	173 (7.2)	73 (5.1)	
	3-4	216 (9.2)	197 (8.2)	71 (4.9)	
	5-6	119 (5.1)	100 (4.1)	37 (2.6)	
	≥7	290 (12.4)	288 (12.0)	117 (8.1)	
Variety of SA participation (activity)					<0.001
	0	379 (16.2)	672 (27.9)	614 (42.8)	
	1	1,302 (55.5)	1,286 (53.4)	671 (46.7)	
	≥2	665 (28.3)	449 (18.6)	151 (10.5)	
Frequency of participation in SA		7.53±5.20	6.42±5.34	5.22±5.39	<0.001
Smoking status					<0.001
	Current smoker	779 (33.2)	707 (29.4)	375 (26.1)	
	Other	1,567 (66.8)	1,700 (70.6)	1,061 (73.9)	
Alcohol consumption					<0.001
	Current drinker	1,170 (49.9)	1,007 (41.8)	496 (34.5)	
	Other	1,176 (50.1)	1,400 (58.2)	940 (65.5)	

Values are presented as number (%) or mean±standard deviation. SA, social activity.

1 Using the chi-square test and analysis of variance for categorical and continuous covariates, respectively.

**Table 3.** Intercepts and slopes of the cognitive trajectories

Variables	Total	Young-old	Middle-old	Old-old
Intercept mean	24.32 (24.18, 24.45)	26.86 (26.72, 26.99)	24.43 (24.24, 24.63)	19.97 (19.62, 20.32)
Slope mean	-0.20 (-0.21, -0.18)	-0.09 (-0.11, -0.07)	-0.20 (-0.23, -0.17)	-0.44 (-0.49, -0.38)
Intercept variance	25.02 (23.91, 26.13)	7.89 (7.23, 8.54)	18.31 (16.93, 19.68)	34.58 (31.11, 38.06)
Slope variance	0.17 (0.16, 0.19)	0.09 (0.08, 0.10)	0.19 (0.16, 0.21)	0.29 (0.22, 0.37)
Intercept-slope covariance	0.06 (0.00, 0.11)	-0.14(-0.22, -0.06)	-0.10 (-0.17, -0.02)	-0.08 (-0.21, 0.05)

Values are presented as β regression coefficient (95% confidence interval).

**Table 4.** Effects of lifestyle factors on intercepts and slopes among age group^1^

Variables		Total		Young-old		Midd-old		Old-old	
		Intercept	Slope	Intercept	Slope	Intercept	Slope	Intercept	Slope
Lifestyle factors									
	Regular exercise	0.30 (-0.20, 0.79)	-0.06 (-0.13, 0.01)	-0.28 (-0.77, 0.21)	0.01 (-0.07, 0.08)	0.95 (0.17, 1.74)	-0.11 (-0.23, 0.01)	-0.56 (-2.12, 1.00)	-0.26 (-0.55, 0.03)
	Regular exercise duration	0.24 (0.11, 0.37)	-0.01 (-0.03, 0.01)	0.24 (0.11, 0.37)	-0.01 (-0.03, 0.01)	0.10 (-0.11, 0.31)	-0.01 (-0.04, 0.02)	0.43 (0.01, 0.85)	0.03 (-0.04, 0.11)
	Variety of SA participation	0.07 (-0.19, 0.33)	0.00 (-0.03, 0.04)	-0.13 (-0.39, 0.13)	0.00 (-0.04, 0.04)	0.17 (-0.25, 0.58)	-0.02 (-0.08, 0.05)	0.67 (-0.17, 1.51)	0.05 (-0.10, 0.19)
	Frequency of SA participation	0.19 (0.14, 0.24)	0.01 (0.00, 0.02)	0.14 (0.08, 0.20)	0.01 (-0.00, 0.02)	0.13 (0.06, 0.21)	0.02 (0.01, 0.03)	0.17 (0.04, 0.30)	-0.01 (-0.03, 0.02)
	Smoking status	0.53 (0.22, 0.84)	-0.05 (-0.09, -0.00)	0.19 (-0.15, 0.54)	-0.05 (-0.11, -0.00)	0.99 (0.51, 1.47)	-0.04 (-0.11, 0.04)	0.55 (-0.24, 1.35)	-0.10 (-0.26, 0.06)
	Alcohol consumption	0.01 (-0.26, 0.28)	-0.01 (-0.05, 0.03)	0.27 (-0.02, 0.56)	-0.03 (-0.08, 0.00)	-0.22 (-0.66, 0.21)	-0.01 (-0.08, 0.06)	-0.44 (-1.17, 0.29)	0.04 (-0.11, 0.18)
Covariates									
	Age	-1.99 (-2.16, -1.81)	-0.14 (-0.17, -0.12)	-0.05 (-0.10, -0.01)	-0.01 (-0.02, -0.00)	-0.23 (-0.29, -0.17)	-0.02 (-0.03, -0.01)	-0.40 (-0.47, -0.34)	-0.03 (-0.04, -0.01)
	Sex	0.36 (0.02, 0.69)	0.03 (-0.02, 0.08)	-0.07 (-0.44, 0.30)	0.07 (0.01, 0.12)	0.32 (-0.21, 0.85)	0.02 (-0.06, 0.10)	1.82 (0.90, 2.73)	0.09 (-0.09, 0.27)
	Level of education	1.48 (1.21, 1.74)	0.01 (-0.03, 0.05)	1.18 (0.91, 1.45)	0.01 (-0.03, 0.05)	1.53 (1.12, 1.94)	0.00 (-0.06, 0.07)	2.25 (1.42, 3.07)	-0.03 (-0.19, 0.12)
	Marital status	1.33 (1.05, 1.61)	0.00 (-0.04, 0.04)	-0.13 (-0.50, 0.25)	0.01 (-0.05, 0.07)	0.56 (0.14, 0.98)	0.00 (-0.07, 0.07)	1.09 (0.37, 1.81)	-0.08 (-0.21, 0.05)
	Area of residence	0.23 (-0.03, 0.48)	0.01 (-0.03, 0.04)	-0.05 (-0.34, 0.24)	0.04 (0.00, 0.09)	0.46 (0.07, 0.86)	0.01 (-0.05, 0.07)	0.48 (-0.16, 1.13)	-0.09 (-0.21, 0.03)
	Log-transformed household income	-0.13 (-0.22, -0.03)	0.02 (0.01, 0.03)	0.08 (-0.05, 0.22)	0.02 (0.00, 0.04)	-0.04 (-0.18, 0.10)	0.02 (-0.01, 0.04)	-0.09 (-0.28, 0.09)	0.02 (-0.02, 0.06)
	Employment status	0.68 (0.41, 0.95)	-0.01 (-0.05, 0.03)	0.13 (-0.14, 0.41)	0.00 (-0.04, 0.04)	1.02 (0.60, 1.43)	-0.04 (-0.11, 0.02)	1.48 (0.45, 2.52)	-0.02 (-0.20, 0.15)
	Chronic diseases	0.18 (0.04, 0.33)	-0.02 (-0.04, 0.01)	-0.20 (-0.37, -0.03)	0.01 (-0.02, 0.04)	-0.14 (-0.37, 0.08)	-0.01 (-0.05, 0.02)	0.58 (0.20, 0.95)	-0.04 (-0.11, 0.03)
	Self-rated health	0.75 (0.60, 0.90)	0.00 (-0.02, 0.03)	0.40 (0.24, 0.57)	0.00 (-0.02, 0.03)	0.73 (0.49, 0.96)	0.00 (-0.04, 0.04)	1.07 (0.69, 1.45)	0.04 (-0.04, 0.11)
	Depressive symptom	-0.26 (-0.30, -0.22)	0.01 (0.00, 0.01)	-0.20 (-0.24, -0.15)	0.01 (0.00, 0.02)	-0.23 (-0.29, -0.17)	0.01 (-0.00, 0.02)	-0.35 (-0.45, -0.24)	0.01 (-0.01, 0.03)

Values are presented as β regression coefficient (95% confidence interval). SA, social activity.

1 The presented model is adjusted for age group, sex, education, marital status, residence, log-transformed household income, employment status, chronic diseases, self-rated health, and depressive symptoms; The crude model is shown to improve table readability.
